# Measurements of large optical rotary dispersion in the adipose eyelid of Atlantic mackerel (*Scomber scombrus*)

**DOI:** 10.1098/rsif.2023.0025

**Published:** 2023-04-05

**Authors:** Euan Jenkinson, Andrew J. Alexander, Philip J. Camp

**Affiliations:** School of Chemistry, University of Edinburgh, David Brewster Road, Edinburgh EH9 3FJ, Scotland

**Keywords:** polarization, optical rotatory dispersion, mackerel

## Abstract

Collagen is the most prevalent of Nature’s structural proteins, and is found in the extracellular matrices of animals. The structures of collagen molecules and aggregates are chiral, which leads to the rotation of transmitted, plane-polarized light. Here, it is shown that the concentrations of chiral molecules and aggregates in the optically transparent, adipose eyelid of Atlantic mackerel (*Scomber scombrus*) can be so high, that plane-polarized light in the visible spectrum is rotated by tens to hundreds of degrees, depending on wavelength (the optical rotatory dispersion (ORD)). This gives rise to intensely coloured images of eyelid samples when illuminated with white light and viewed between crossed polarizers. The ORD in the visible spectrum is measured with monochromatic light sources, and using this dispersion, the variation of optical thickness within a sample (proportional to collagen concentration and path length) is determined. The agreement between observed and simulated white-light images is almost perfect. While collagen provides vital mechanical rigidity to animal tissue, it might also possess optical properties that are useful for vision and camouflage.

## 1. Introduction

**Figure 1.**
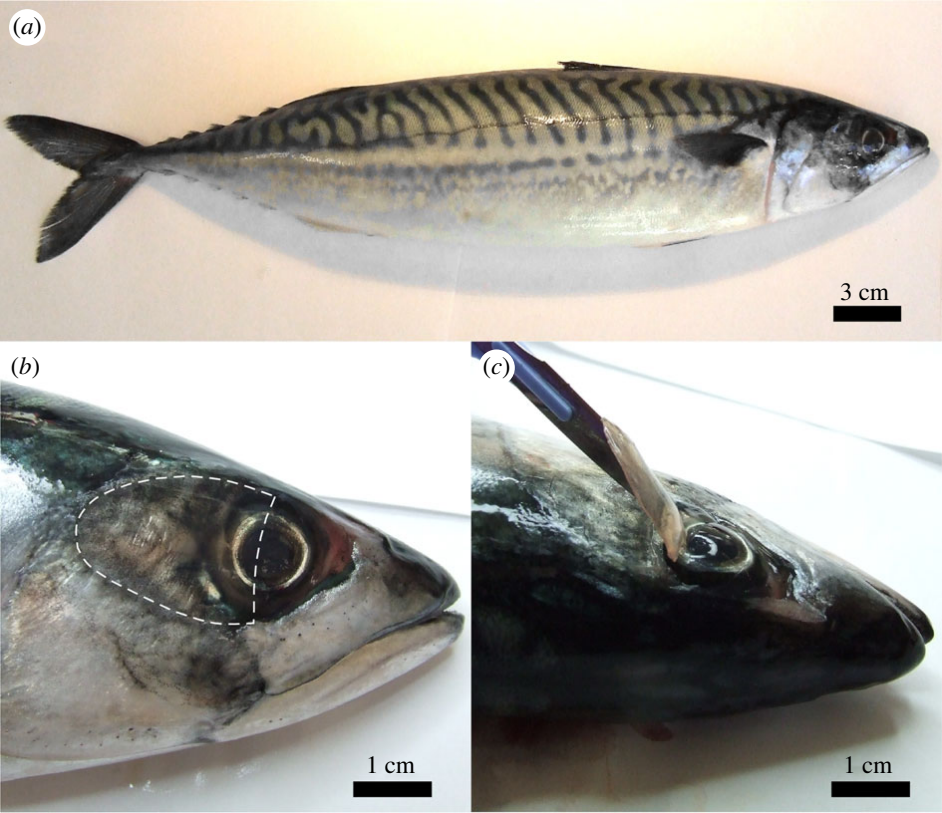
Images showing (*a*) Atlantic mackerel (*Scomber scombrus*); (*b*) location of the adipose eyelid; (*c*) a sample of adipose eyelid after removal by scalpel.

Many freshwater and marine fishes have highly reflective skin, with or without large scales, which acts as camouflage against predators. Open-water, top-feeders like mackerel are silver on their ventral side, and dark on their dorsal side, in order to avoid detection from below and above, respectively; see figure 1*a*. The high reflectivity of fish skin is due to the presence of micrometre-sized, guanine crystals, which are stacked in the stratum argentum of the dermis, resulting in an efficient, multi-layer reflector [1–6]. As much as 80% of the incident light undergoes specular reflection. By contrast, the skin of shallow-water, bottom-feeder fishes like eels produces more diffuse reflection [7].

As well as the intensity of light, the polarization of light may play an important role in animal vision [6]. When discussing polarization vision, three quantities are relevant to the observer: (i) the degree of polarization, (ii) the angle of polarization, and (iii) the ellipticity [8]. The degree of polarization is a measure of how polarized is the light. For unpolarized light, the direction of the electric field vector (***E***) is random in space and time. If ***E*** has a preferred axis of alignment with respect to its direction, this can be quantified by the polarization angle. Ellipticity describes the extent to which ***E*** rotates as it travels, the extremes being left and right circularly polarized light, with linearly polarized light in between.

**Figure 2.**
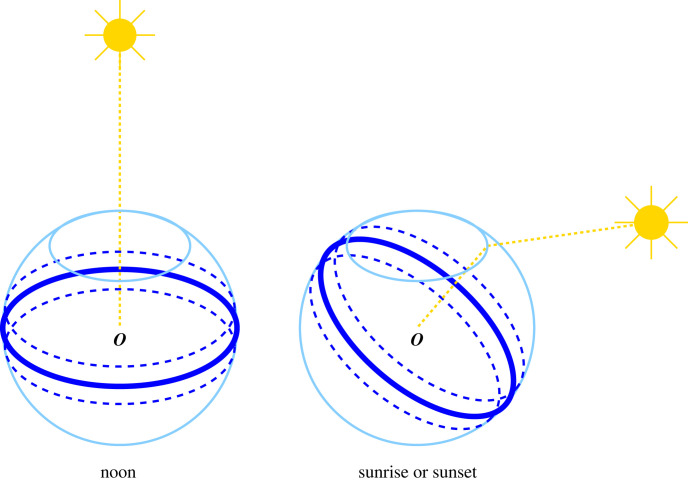
Schematic diagram illustrating the polarization of submarine light, adapted from fig. 3 of [10]. Observer ***O*** is several metres underwater. The Sun’s light is shown by gold dotted lines. The light underwater is polarized according to where the observer looks on a sphere (light blue), truncated by the ocean’s surface, and with the observer at the centre. At noon, when the Sun is near the zenith, the *E*-vector of the light is horizontally polarized in all directions, and the maximum polarization is found on the great circle (dark blue solid line), and within a band either side (dark blue dashed lines). At sunrise or sunset, the band of maximum polarization is still perpendicular to the light from the Sun, which is refracted at the air–water interface, and the polarization is more vertical.

The submarine polarization field of light differs from the terrestrial polarization field due to scattering by molecules [9–11]. A simple schematic diagram adapted from fig. 3 of [10] is presented in figure 2. Relative to an observer, the degree of polarization underwater is maximal in a band of light that is perpendicular to the direction of the Sun, and the angle of polarization is also perpendicular to that direction. When the Sun is directly above, a swimmer encounters horizontally polarized light all around in a horizontal plane, i.e. to the front, back and sides. When the Sun is rising or declining, the band and polarization angles are tilted to be more vertical. The degree of polarization becomes greater at dawn and dusk, but is generally less than 50%. The degree of polarization does not vary much with wavelength from the middle of the near UV to the red end of the visible spectrum (350–600 nm) [11]. Many more details are available from direct experimental measurements [9,11].

**Figure 3.**
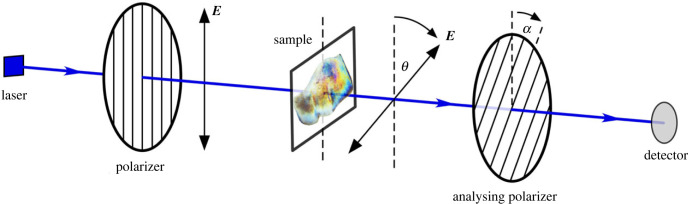
Schematic diagram illustrating the ORD and imaging experiments. Monochromatic light of wavelength *λ* from a laser is passed through a polarizer: on passing through the eyelid sample the plane of polarization is rotated by angle *θ*; the angle of polarization *θ* is measured by rotating an analysing polarizer by angle *α*; the signal measured at the photodiode detector exhibits a cosine-squared dependence, and is maximal when *α* = *θ*.

The skin of many fishes is highly reflective. In general, regular (specular) reflections significantly increase the degree of polarization of the reflected light. Lythgoe and Hemmings showed that by altering the orientation of a plane polarizer, reflective surfaces—including the skin of fishes—can appear brighter when imaged underwater [12]. Jordan *et al.* investigated the polarization dependence of the reflections as a function of direction and wavelength [13]. They found that birefringent guanine crystals can align with the low-refractive index axis pointing either normal to, or in the plane of, the dermis (type 1 and type 2, respectively). These two populations stack in combination, and result in a low degree of polarization, independent of direction of observation or wavelength. As Jordan *et al.* explained, the arrangement neutralizes the usual polarizing effect of reflections. The mechanism for reflectivity of fish skin has evidently evolved to match the background intensity of light underwater. It is not clear whether mechanisms that neutralize polarization stem from evolutionary pressure, or are simply coincidental. The current understanding of the role of light polarization in animal vision has been reappraised recently by Marshall and co-workers [14]. Roberts identifies three themes underlying polarization vision—mechanisms, neural processing and animal behaviour—and points out that retinal structure is a potential indicator for polarization sensitivity [15]. It is well known that the eyes of octopuses have structures which can distinguish between horizontally and vertically polarized light [16]. In fishes, structure capable of sensing polarization has been seen in the retinas of anchovies, for example, and it has been shown that some species of fish are more sensitive to UV (380 nm) polarization [10]. The reason for polarization sensitivity—if there is one—is not known [17]. Johnsen *et al.* have shown that polarization sensitivity is unlikely to increase the range of vision [18]. Venables *et al.* suggest that polarization sensitivity may help reduce the negative effects of visual noise (flickering) arising from caustics [19]. It has been shown in field measurements that the skin of open-ocean fishes both reflects and polarizes incident light, while that of species in nearshore, depolarized-light environments only reflects [20]. There are even open-water fishes that appear to alter their polarization properties depending on the time of day and the position of the Sun [13,21,22]. It is therefore tempting to speculate that open-water fishes have developed camouflage to avoid detection by predators *whose vision is sensitive to polarization*.

Some fishes possess external structures around the eye that may serve some sort of optical function. Adipose eyelids—so-called because they were originally thought to be ‘fatty’—are transparent, fully cover the eyes of deep-sea fishes, and partially cover the eyes of some other species, including herring, mackerel, milkfish and mullet [23]. The eyelids comprise epithelial tissue surrounding collagen fibrils [24,25]. The functions of the eyelids may include providing mechanical protection to the eye, reducing hydrodynamic drag around the protruding eyeball [24,25], being a light filter (based on wavelength), and acting as a focusing lens [26]. In some cases, the adipose eyelid is birefringent, and it may act as a polarizing filter to aid vision [26], although this was shown not to happen in the particular case of sockeye salmon [27].

Herein, the optical properties of the adipose eyelid of Atlantic mackerel (*Scomber scombrus*) are investigated experimentally and with a simple model. While studying the reflectivity of mackerel skin, it was noticed accidentally that the thick (approx. 2 mm), transparent layer of gel-like material immediately surrounding the posterior side of the eye produces significant rotation (approx. $10^{\circ }$–$100^{\circ }$) of polarized visible light. Adipose eyelids contain collagen [24,25], and while its molecular chirality gives rise to optical rotation [28–30], it also forms bundles of chiral self-assembled fibrils and fibres [31] which exhibit complex optical properties [32]. The amount of rotation depends on the *optical thickness* of the material (which is proportional to the concentration of chiral species and structures, and the path length) and the wavelength of the light. The wavelength dependence is called optical rotatory dispersion (ORD) [33]. The presence of strong ORD is most easily demonstrated by imaging the material between crossed polarizers [34–36]; the angle between the crossed polarizers dictates which wavelengths of light are transmitted by the first polarizer, rotated by the material, transmitted through the second polarizer, and subsequently imaged. ORD can also be characterized by measuring the fluorescence emission spectrum of a chiral medium [37]. Strong ORD means that the rotation angle depends strongly on wavelength, and therefore, the colours transmitted by the second polarizer change significantly with polarizer angle. In the current experiments, the optical thickness and ORD of the adipose eyelid are so strong, that intense colours are observed when imaged in this way. The ORD is determined by using almost monochromatic light sources, and the results are incorporated in a simple model that simulates the effects of the material with a given optical thickness (strength of rotation) and ORD (dependence on wavelength). It is possible to take an optical image, determine the optical thickness pixel-by-pixel, and thereby reveal possible structural and/or growth patterns in the material. The accuracy of the model is demonstrated by comparing simulated and experimental images, which are almost indistinguishable. Some speculations on the biological role of the strong ORD are put forward.

## 2. Methods

Fresh Atlantic mackerel (*Scomber scombrus*) were obtained from local fishmongers. Eyelid samples were removed using a scalpel, as illustrated in figure 1*b*,*c*. The size and shape of the excised portion varies roughly according to the size of the fish. The samples were transferred to a salt solution composed of 3.6% w/w NaCl in deionized water (Fisher, HPLC grade). All fish and eyelid samples were stored in a refrigerator ($4^{\circ }C$) while not in use.

An outline of the experimental set-up for measuring ORD is shown in figure 3. More technical details are given in figure S1 of the electronic supplementary material. Incident light—either from a laser or a white-light source—was passed first through a linear polarizer. This ensured that the plane of polarization, defined by the direction of the oscillating electric field of the light (***E***), was known. After passing through the sample, the plane of polarization of each wavelength of light (*λ*) had been rotated by an angle *θ*(*λ*). An analysing polarizer at an angle *α* with respect to the first polarizer preferentially transmitted those components of light that had been rotated by the same angle. In order to avoid stress on the tissue sample, a vertical arrangement of the optical components was used, and the sample was laid flat in a dish containing the salt solution. Two types of experiments were conducted. With a white-light source and a digital camera, colour images of the sample were obtained at various analysing-polarizer angles *α*. With the white-light source and digital camera replaced by a continuous-wave laser and photodiode, respectively, the polarization-rotation angle *θ* as a function of wavelength *λ* could be determined.

With a white-light source, and a digital camera imaging the transmitted light, the sample showed a broad range of colours, depending on the local optical thickness within the sample (which controls the degree of rotation), and the analysing-polarizer angle *α* (which controls which components of the transmitted light are detected). The camera was used to identify small regions of the sample (approx. 1.5 mm^2^) that exhibited a broad range of intense, homogeneous colours with varying *α*, indicating strong ORD, and high and uniform optical thickness. A continuous-wave laser was then put in place of the white-light source, and an iris was placed in the beam path to ensure that only the selected portion of sample was exposed to the incident, monochromatic, polarized light. To determine *θ* for each wavelength *λ*, the analysing polarizer was rotated through a series of angles *α* from $0^{\circ }$ to $360^{\circ }$ in steps of $10^{\circ }$, and the intensity of light *I*(*α*) reaching a photodiode (in place of the digital camera) was measured. The power of the incident laser light was adjusted carefully to avoid saturating the photodiode. Note that both white-light and discrete-wavelength sources are used in this study, but that for quantitative measurements of ORD (and similar phenomena, such as the magneto-optical Faraday effect), the use of discrete wavelengths is less prone to systematic errors [38].

White-light images and ORD measurements were obtained for a large number of samples. For each sample, a region was selected for ORD measurements, as indicated above. The results for all samples were qualitatively similar, although the strength of the ORD, and the intensity of the observed colours, varied. Sample 1 is discussed in detail in the Results section, and the results for sample 2 are presented in the electronic supplementary material. Sample 1 showed more intense colours than sample 2 when viewed through crossed polarizers, and so sample 2 provided a stringent test of the analysis and simulation methods.

## 3. Results

### 3.1. White-light images

**Figure 4.**
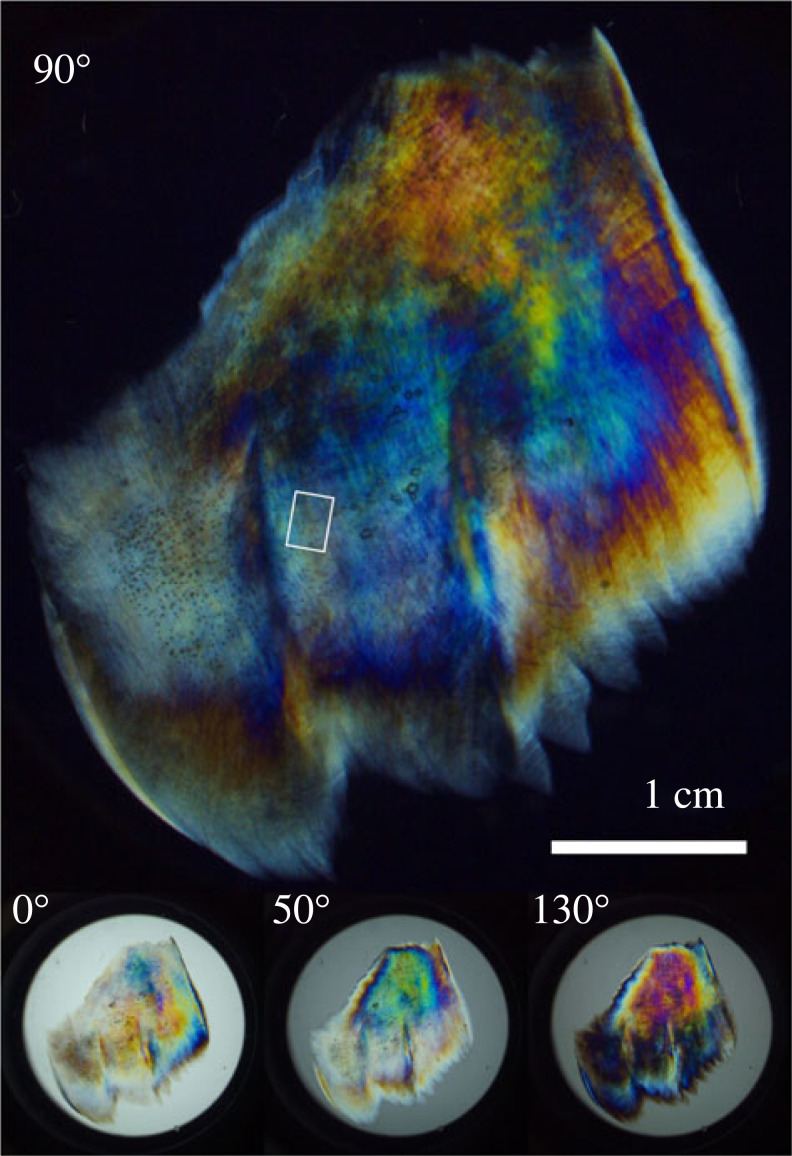
Colour images of sample 1 demonstrating the ORD of adipose eyelid sample from Atlantic mackerel through crossed polarizers as a function of the angle of rotation of the analysing polarizer (figure 3). The area of eyelid sample analysed is indicated in the image at $90^{\circ }$. The sample is oriented such that the part nearest the eye is along the right-hand edge.

Figure 4 shows white-light images of a piece of mackerel eyelid of width approximately 3 cm. Images are shown for analysing-polarizer angles of $0^{\circ }$, $50^{\circ }$, $90^{\circ }$ and $130^{\circ }$. The full set of images at intervals of $10^{\circ }$ is provided in figure S3 of the electronic supplementary material. Firstly, the circular background shows that the intensity of transmitted white light was maximal at $\alpha =0^{\circ }$ (equivalent to $180^{\circ }$) and minimal at $\alpha =90^{\circ }$, according to the expected cos ^2^*α* relationship. Secondly, the eyelid sample exhibited many different colours depending on the value of *α*. For example, in the upper half of the sample, there are small blue regions at low angles $\alpha =0^{\circ }$–$10^{\circ }$, growing intensity of blue, green and yellow in the range $\alpha =20^{\circ }$–$70^{\circ }$, a change towards red and then purple in the range $\alpha =80-130^{\circ }$, and finally a reduction in colour again towards $\alpha =180^{\circ }$ (equivalent to $0^{\circ }$). The intensity and dispersion of colours are governed by the optical thickness of the material—related to the non-uniform collagen concentration and path length in the sample—and the ORD of the collagen itself. To explore the latter, a small, optically uniform region of the sample was chosen to be analysed further, and this region is shown in the image for $90^{\circ }$ in figure 4.

### 3.2. Optical rotatory dispersion measurements

For the selected region of the sample, the ORD was determined at five different wavelengths by measuring the voltage signal *S* at the photodiode detector (figure 3) as a function of analysing-polarizer angle *α*. *S* is proportional to the intensity *I* of transmitted light, and hence it was fitted with a function based on Malus’s Law [8]

3.1 $$I(\alpha )\propto S(\alpha )=S_{0}\cos^{2}(\alpha -\theta )+C,$$

where *S*_0_ and *C* are the detector sensitivity and offset voltage parameters, respectively. The offset *C* accounts for any background unpolarized light incident on the detector. The parameter *θ* represents the rotation angle of the transmitted light due to ORD. The analysis of the raw data for each sample is presented in figure S2 of the electronic supplementary material, and the resulting values of *θ* for each wavelength are given in table 1.

**Table 1.** The optical rotation angle *θ* as a function of wavelength *λ*, and the fitted values of the optical thickness *A* and resonance wavelength *λ*_0_ (equation (3.2)), for samples 1 and 2.

	sample 1	sample 2
$\theta (410\;\mathrm{nm})\;{(}^{\circ })$	119 ± 1	26 ± 3
$\theta (488\;\mathrm{nm})\;{(}^{\circ })$	59 ± 3	18 ± 1
$\theta (532\;\mathrm{nm})\;{(}^{\circ })$	33 ± 1	12 ± 2
$\theta (635\;\mathrm{nm})\;{(}^{\circ })$	32 ± 2	6 ± 3
$\theta (670\;\mathrm{nm})\;{(}^{\circ })$	28 ± 1	8 ± 2
$A\;{(}^{\circ })$	63 ± 19	62 ± 46
*λ*_0_ (nm)	331 ± 18	227 ± 65

**Figure 5.**
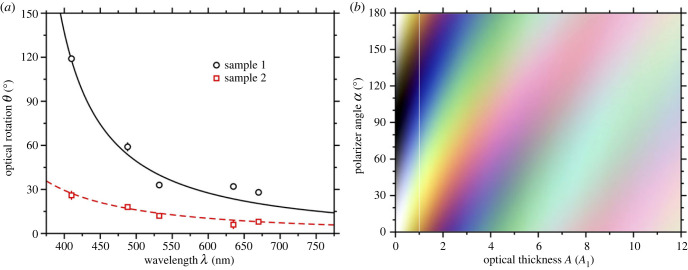
(*a*) Plot of the dependence of ORD rotation angle (*θ*) on the wavelength of incident light (*λ*). Experimental data are represented by symbols, and fits to the experimental data by the Drude equation (equation (3.2)) are shown as lines. The results show a decrease in *θ* with increasing wavelength. (*b*) A colour map showing the effect of ORD on incident white light transmitted through a sample with a prescribed optical thickness, expressed in units of *A*_1_ for sample 1 (table 1), between polarizers crossed at an angle *α*. The vertical white line indicates *A* = *A*_1_. With no ORD (no optical thickness) the transmitted light is on a greyscale with intensity proportional to cos^2^*α*. With very strong ORD (high optical thickness), the light is scrambled into pink and turquoise. With intermediate ORD (approx. *A* = 3*A*_1_) a broad range of colours is observed as the polarizer angle is varied.

Figure 5*a* shows a plot of the value of *θ* obtained at each wavelength used, and for samples 1 and 2. The rotation angles for sample 2 were significantly lower than those for sample 1, but together these are representative of the ORD observed in different samples. The dependence of *θ* on *λ* was modelled using the Drude equation [33]

3.2 $$\theta (\lambda )=\frac{A\lambda_{0}^{2}}{\lambda^{2}-\lambda_{0}^{2}},$$

where *A* is the optical rotary power parameter, i.e. optical thickness, and *λ*_0_ is the characteristic (resonance) wavelength. *A* is proportional to the concentration of chiral material, and the path length. The remaining factors in this equation capture the wavelength variation of the specific rotation. The fitted values of *A* and *λ*_0_ are given in table 1. The apparent resonance wavelengths vary substantially, with that for sample 2 being much lower than that for sample 1. The fitting errors in *λ*_0_ are 5–29%, while the deviation of each value from the mean is 19%, and so the apparent difference between the samples is not so significant. Since the optical thicknesses are similar, the resonance wavelength accounts for the lower measured rotation angles for sample 2. The resonance wavelength for sample 2 is typical of that for collagen, being in the region of 200 nm [28,30]. Self-assembled fibrils and fibres of collagen in the sample [24,25,32] will have different, and presumably greater, optical activity than free collagen molecules, and in addition, there may be other chiral molecules, proteins and aggregates present in the sample. The high rotation angle measured at short wavelengths in sample 1 accounts for the substantially higher fitted value of *λ*_0_. In any case, it is sufficient that the fitted functions describe the measured data adequately in the visible region of the spectrum.

### 3.3. Analysis of white-light images

To establish the link between the white-light images and the ORD measurements, a colour map was produced. For a given polarizer angle *α*, the intensity of light of wavelength *λ* transmitted through a *homogeneous* region (or pixel) with optical thickness *A* is given by

3.3 $$I(\lambda )\cos^{2}\left(\alpha -\frac{A\lambda_{0}^{2}}{\lambda^{2}-\lambda_{0}^{2}}\right),$$

where *I*(*λ*) is the intensity of incident light. If the incident visible-light spectrum is represented by a vector of RGB values ***I***(*λ*) = (*R*_*I*_(*λ*), *G*_*I*_(*λ*), *B*_*I*_(*λ*)), where 380 nm ≤ *λ* ≤ 780 nm [39], then the transmitted RGB values ***M*** are given by the vector

3.4 $$\begin{array}{rl} M & =(R_{M},G_{M},B_{M})\\ & =\int_{380\;\mathrm{nm}}^{780\;\mathrm{nm}}I(\lambda )\cos^{2}\left(\alpha -\frac{A\lambda_{0}^{2}}{\lambda^{2}-\lambda_{0}^{2}}\right)d\lambda . \end{array}$$

Note that similar approaches have been described in the education literature for analysing colour images arising from ORD [40]. Figure 5*b* shows a colour map, where the optical thickness in the interval [0, *A*_max_] is on the abscissa, the polarizer angle $0\leq \alpha \leq 180^{\circ }$ is on the ordinate, and the optical thickness *A* = *A*_1_ is highlighted with a vertical white line. For visualization purposes, the RGB values at each angle for a given optical thickness were rescaled by the same factor so that they span the full range [0, 255]. The ORD function (equation (3.2)) used to generate figure 5*b* was that for sample 1, and *A*_max_ = 12*A*_1_, where *A*_1_ is the optical thickness. The colour map shows several interesting features. First, with zero optical thickness, the transmitted light is on a simple grey scale, with white-light intensity proportional to cos^2^*α*. Second, with a large optical thickness (10 times that of sample 1), the polarization angles of all wavelengths are rotated to such a degree, that there is a smearing out of colour, and it is only possible to identify pink regions at $\alpha ≃0^{\circ }$ and $180^{\circ }$, and a turquoise region at $\alpha ≃90^{\circ }$. These bands shift in *α* with increasing optical thickness. Third, with an intermediate optical thickness (approx. three times that for sample 1) distinct colours can be observed. This means that the rotation angles (*modulo* $180^{\circ }$) of the light at each wavelength are well spread out in the range from $0^{\circ }$ to $180^{\circ }$, and that it is therefore possible to ‘pick out’ certain bands of wavelengths by changing the analysing-polarizer angle *α*.

The experimental photographs were simulated by determining the effective optical thickness *A* pixel-by-pixel. First, the experimental JPEG image for a given value of *α* was converted to RGB format, giving a vector ***P*** = (*R*_*P*_, *G*_*P*_, *B*_*P*_) for each pixel, and the corresponding relative luminance was calculated on a 0–255 scale using *L*_*P*_ = 0.2126*R*_*P*_ + 0.7152*G*_*P*_ + 0.0722*B*_*P*_ [41]. Next, a colour map was generated for the given value of *α*—as in figure 5*b*—and on a fine grid of optical thicknesses in the range [0, *A*_max_], where *A*_max_ is some multiple of the fitted optical thickness for the analysed region in a particular sample. For each pixel in the experimental image, the RGB values in the colour map ***M*** were rescaled to the same relative luminance, giving ***Q*** = ***M****L*_*P*_/*L*_*M*_, where *L*_*M*_ is the colour-map relative luminance. Finally, the apparent optical thickness was identified as the one that minimized |***P*** − ***Q***|^2^, and a new image was produced for comparison with the original experimental image.

**Figure 6.**
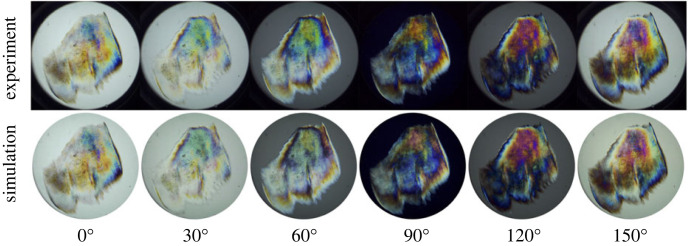
Comparison between experimental (top row) and simulated (bottom row) white-light images at selected polarizer angles, using the ORD curve for sample 1.

Figure 6 shows a direct comparison between experimental and simulated images for sample 1, at angles $\alpha =0^{\circ }$–$150^{\circ }$ in $30^{\circ }$ intervals; in this case, *A*_max_ = 12*A*_1_ was sufficient to reproduce the full range of colours, where *A*_1_ is given in table 1. The top row shows the experimental white-light images, and the bottom row shows the simulated white-light images. The agreement between experiment and simulation is, by eye, almost perfect. There are some minor deviations, for example at $\alpha =60^{\circ }$, where the blue and green shades are not as intense in the simulated images, but it is difficult to tell the experimental and simulated images apart. Overall, the agreement is such that the model, and the measured ORD in figure 5*a*, provide a complete description of the white-light images.

**Figure 7.**
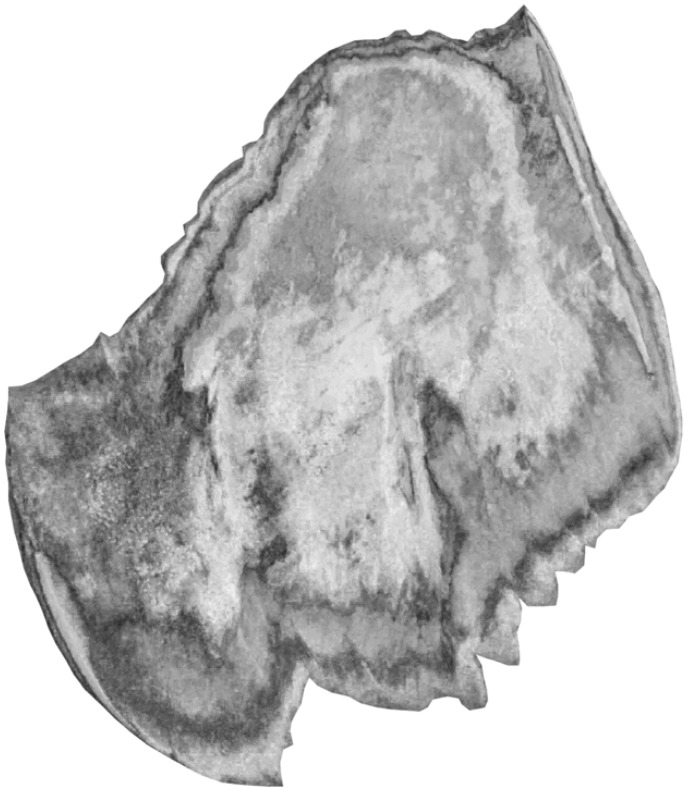
Average optical-thickness map *A*(*x*, *y*) corresponding to figure 6. The optical thickness is shown on a grey scale, where black corresponds to an optical thickness equal to *A*_max_ = 12*A*_1_, and white means *A* = 0.

Finally, figure 7 shows the corresponding average optical-thickness map *A*(*x*, *y*) on a grey scale, where black corresponds to an optical thickness equal to *A*_max_ = 12*A*_1_, and white means zero optical thickness. Roughly speaking, the biggest variations in colour in figures 4 and 6 correlate with ‘medium’ optical thickness, relative to *A*_max_ = 12*A*_1_; this corresponds to the region *A* ≃ 3*A*_1_ in the colour map in figure 5*b*. Very high optical thickness scrambles the polarization at all wavelengths, and this means that the intensity of colour is low.

The optical-thickness map in figure 7 indicates a heterogeneous distribution of chiral material throughout the eyelid sample. There appear to be at least two layers, with high optical thickness at the edges. Atlantic mackerel can live for up to 17 years, and reach sexual maturity after 2 years [42]. It is possible that the layers in the eyelid sample correspond to different stages of growth, possibly as a result of seasonal cycles, or of feeding activity linked to the abundance of prey fish.

Figures 5*b*–7 were generated using the ORD relation measured for sample 1. The corresponding results using the ORD relation measured for sample 2 are given in electronic supplementary material: figure S2 shows the analysis of the ORD; figure S4 shows all of the experimental and simulated white-light images; figure S5 shows the colour map; and figure S6 shows the optical-thickness map. The analysis was carried out in exactly the same way, except that *A*_max_ = 18*A*_2_, because the rotation angles were smaller than for sample 1 (figure 5*a*). The agreement between experiment and simulation is again almost perfect.

## 4. Conclusion

The results of this optical and simulation study show that the millimetre-thick, optically transparent, adipose eyelid of Atlantic mackerel can contain sufficient concentrations of chiral material—such as collagen—to rotate the polarization of visible light by tens to hundreds of degrees. This phenomenon can be observed in the laboratory as an array of intense colours when the sample is imaged between crossed polarizers, and illuminated by white light. The colours and the variation with polarizer angle vary throughout the sample, and depend on the local optical thickness of chiral material (proportional to the concentration and path length). The measured ORD within optically uniform regions of the sample can be incorporated into a simple model, which reproduces the experimental images almost perfectly. The model shows how the optical thickness varies throughout the sample, and the results indicate that there are distinct layers, perhaps connected with the growth of the fish.

That the model and the experiment are in almost perfect agreement shows that the basic physics of the effect is understood. But does ORD have any biological function? Collagen provides tensile strength to the tissue, and is chiral, and these two properties could be unrelated. But it is interesting that the optically transparent material is so thick immediately behind the eye. As noted in the Introduction, the optical properties of the adipose eyelid could confer some *accidental* aid to vision, and when considering the role of light polarization in animal vision, the birefringence and polarization-filtering properties could be beneficial. But in this work, it has been shown that incident light polarization is completely scrambled by the high optical thickness and strong ORD of the eyelid, and so if that confers an optical advantage, then it must be different from those considered before.

As explained in the Introduction, the light underwater is partially polarized. When the light undergoes specular reflection from e.g. the reflective scales of a prey fish, the polarization is at least partially conserved [2]. Mackerel hunt their prey in the topmost layers of the sea. If the eye of the mackerel possesses a polarization filter, then reflective fish scales will appear brighter against the background [12], which consists of a large region of water with a long optical path length, and consequently transmits scattered and unpolarized light. An analogy could be the difference between greyscale and colour images, where the colour information can be used to distinguish between objects with the same light intensity. In the case of mackerel, perhaps the polarization plays the role of colour. When mackerel are hunting prey, the most important direction of vision is forward, and so perhaps the thick layer of light-rotating material behind the eye helps to enhance the contrast between vision in the forward and backward directions. To use another analogy, this could be like putting blinkers on a horse to avoid it being distracted from what is going on behind it.

## Data Availability

The data supporting this article are provided in electronic supplementary material [43].
